# Proton transfer reagent cations for ion–ion charge state manipulation of high mass negatively-charged analytes in an electrodynamic ion trap

**DOI:** 10.1039/d5an01354b

**Published:** 2026-02-18

**Authors:** Nicholas R. Ellin, Boukar K. S. Faye, Seth A. Horn, Alexander M. Koers, Scott A. McLuckey

**Affiliations:** a Department of Chemistry, Purdue University 560 Oval Drive West Lafayette IN 47907-2084 USA mcluckey@purdue.edu nellin@purdue.edu faye@purdue.edu horn96@purdue.edu koers@purdue.edu +1 (765) 494-0239 +1 (765) 494-5270

## Abstract

This work summarizes the criteria for a useful reagent for ion/ion proton transfer in an electrodynamic ion trap with specific emphasis on proton transfer to high mass multiply-charged analyte ions. A readily available and ionizable weak base of relatively high mass meets the criteria. The protonated form of an extensively fluorinated 10-carbon primary amine (1*H*,1*H*,2*H*,2*H*-perfluorodecylamine, PFDA) is demonstrated to serve as a useful reagent for proton transfer to negatively-charged proteins and oligonucleotides. The behavior of protonated PFDA is compared with that of protonated 1,8-bis(dimethylamino)naphthalene (proton sponge) with respect to the tendency for proton transfer *versus* attachment in ion/ion reactions with a common multiply-deprotonated protein and a common multiply-deprotonated oligonucleotide. Protonated PFDA showed a lesser tendency for ion attachment than the proton sponge in all cases. Both reagent cations showed a greater extent of attachment to the oligonucleotide anions. Mild collisional heating was shown to be able to extensively remove adducted PFDA from the oligonucleotide. The ability to generate low *z* and high *m*/*z* analyte product ions from highly charged precursor analyte ions using protonated PDFA is illustrated with anions derived from β-galactosidase, GroEL, and 30S *E. coli* ribosome particles.

## Abstract 2

This work summarizes the criteria for a useful reagent for ion/ion proton transfer in an electrodynamic ion trap with specific emphasis on proton transfer to high mass multiply-charged analyte ions.

## Graphical abstract

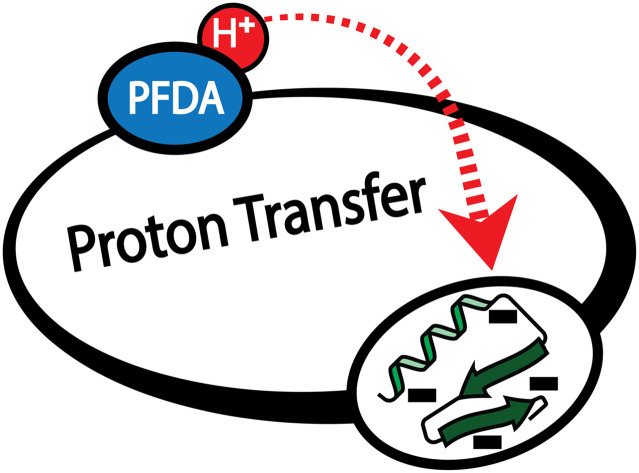

## Introduction

Electrospray ionization (ESI),^1,2^ including its many variations that generate ions from charged droplets, is characterized by its tendency to generate distributions of charge states from large molecules with multiple polar sites,^3^ such as proteins,^4,5^ protein complexes,^6^ oligonucleotides,^7^ polysaccharides,^8^ and some synthetic polymers.^9^ The multiple-charging phenomenon gives rise to both useful and problematic consequences. Among the useful consequences is the ability to determine mass using mass analyzers with relatively modest upper mass-to-charge ratio (*m*/*z*) limits, the ability to determine ion charge *via* the presence of two or more peaks with known differences in mass and charge,^10^ improved resolution for mass analyzers that show a decrease mass resolution as the *m*/*z* ratios increase (*e.g.*, Fourier transform ion cyclotron resonance mass spectrometry^11^ and electrostatic ion traps, such as the Orbitrap^12^), and greater detector response as charge increases.^13^ As the favored dissociation channels for a given molecule are often charge state dependent, the generation of ions over a range of charge states can allow for greater sequence coverage *via* the dissociation of precursor ions over a range of charge states.^14^ On the other hand, multiple-charging compresses ions over a range of masses into a narrower range of *m*/*z*, which can lead to significant overlap of ions of different mass and charge but similar *m*/*z*. This scenario plays out when mixtures of ions, either from completely different analytes or related but heterogeneous analytes, are present. A similar situation can arise from the mixture of product ions generated from the fragmentation of multiply-charged precursor ions.

A variety of approaches have been developed to mitigate or avoid overlap of ions of different mass and charge but the same or similar *m*/*z*.^15^ Charge detection mass spectrometry (CDMS),^16–22^ for example, measures *m*/*z* and *z* simultaneously for a given ion, one or a few ions at a time. CDMS has been shown to be particularly useful for the mass determination of very high mass (*e.g.*, >1 MDa) and heterogeneous species (*e.g.*, virus particles). However, given the time-consuming nature of a CDMS experiment, conventional ensemble measurements, which rely on the measurement of *m*/*z* for many ions more-or-less simultaneously, can generate equivalent or greater ion statistics much more quickly, provided z-state ambiguity is addressed. In order to deal with the *m*/*z* overlap problem in ensemble measurements so that z-states can be determined, a variety of approaches for the manipulation of charge states, usually *via* charge state reduction, have been described. For example, solution additives^23–26^ or exposure of electrospray droplets to acidic,^27^ basic^28^ or organic^29^ vapors can sometimes be used to reduce the charge states generated *via* ESI. An alternative is to subject ESI-generated ions to ion/molecule^30,31^ or ion/ion^32–37^ reactions prior to sampling the ions into the mass spectrometer. Another alternative is to expose analyte ions to ion/molecule,^38,39^ ion/ion,^40–44^ or ion/electron^45,46^ reactions within the mass spectrometer. Charge-state manipulation performed before ions enter the mass spectrometer can only affect ions produced directly by ESI, whereas manipulation performed inside the instrument can be applied to both precursor ions and fragment ions generated during MS/MS. Another benefit of reacting analytes and reagents after introduction to the mass spectrometer is the selectivity afforded by ion isolation of reagent and analyte ions, thereby reducing post-reaction chemical noise.

The use of ion/ion reactions within electrodynamic ion traps has proved to be particularly effective in experiments involving charge state reduction of either precursor,^47,48^ or product ions.^49–55^ The studies cited above involved proton transfer from cationic analytes as the ion/ion reaction mechanism but electron transfer^56^ and ion attachment^57^ have also been demonstrated as means for z-state manipulation in the positive ion mode. Charge state manipulation of multiply-charged anions has not been practiced as often as for multiply-charged cations. In the negative mode, electron photo-detachment^58–61^ and electron-induced electron detachment,^62^ in addition to electron transfer ion/ion reactions^63–68^ have been used for charge-state reduction of anions. Electron ejection/transfer generates a radical site in the analyte, which can, and often does, induce radical-directed dissociation. Proton transfer, on the other hand, involves even-electron products being generated from even-electron precursor ions, which is less likely to lead to fragmentation. Proton transfer to multiply-charged anions in electrodynamic ion traps has been demonstrated^69–73^ using low *m*/*z* reagents, such as protonated isobutylene, protonated pyridine, protonated benzoquinoline, protonated 1,8-bis(dimethylamino)naphthalene (proton sponge) and proton hydrates. The *m*/*z* range over which ions can be stored simultaneously, however, is limited. The *m*/*z* of the reagent, along with several other characteristics, is important in determining its utility for charge state reduction of high mass analyte ions. In this work, we describe a reagent, protonated perfluorodecylamine (PFDA = 463 Da) with favorable characteristics as a reagent for the charge state reduction of high mass protein anions.

## Experimental section

### Materials

Ubiquitin from bovine erythrocytes, 1,8-bis(dimethylamino)naphthalene (proton sponge, PS), tris-HCl, potassium acetate, anhydrous magnesium chloride, ethylenediaminetetraacetic acid (ETDA), Chaperonin 60 (GroEL) from *E. coli*, and β-galactosidase from *E. coli* were purchased from Sigma-Aldrich, St. Louis, MO, USA. *E. coli* ribosome was purchased from New England Biolabs. 1*H*,1*H*,2*H*,2*H*-Perfluorodecylamine (PFDA) was purchased from Biosynth Ltd, UK. 5′-d(A)_30_-3′ (A_30_) DNA was purchased from Integrated DNA Technologies, Coralville, IA, USA. Adenosine-5′-triphosphate (ATP) was purchased from Roche Diagnostics, Mannheim, Germany. Optima LC/MS grade methanol, acetonitrile, acetone, and water were purchased from Fisher Scientific, USA. Amicon Ultra microcentrifuge filters with a 30 kDa and 10 kDa molecular weight cut-offs were purchsed from Millipore, Billerica, MA.

### Analyte preparation

Lyophilized powder of ubiquitin was dissolved in water to create a 117 µM stock solution. The stock solution was then diluted to a final concentration of 4.7 µM with 127 mM ammonium acetate in water to serve as the working solution for negative proton transfer reactions (nPTR).

Lyophilized powder of A_30_ was dissolved in water to create a 1 mM stock solution. The 1 mM stock was further diluted to a final concentration of 4.5 µM of A_30_ with 91 mM ammonium acetate.

GroEL lyophilized powder was dissolved in buffer solution containing 20 mM Tris-HCl, 50 mM potassium acetate, 0.5 mM ETDA, 5 mM magnesium chloride, and 2 mM ATP at pH 7, to a 20 μM concentration by monomer mass. The solution was shaken for one hour at room temperature and diluted to 20% methanol (*v*/*v*) before shaking for another hour at room temperature. Afterwards, the protein was precipitated using 50% acetone, centrifuged and decanted. The protein pellet was re-dissolved in buffer to the starting concentration of 20 µM by monomer mass. It was then concentrated using an Amicon Ultra microcentrifuge filter with a 30 kDa molecular weight cut-off and diluted to 20 µM before shaking for an hour. The concentration, dilution, and shaking steps were repeated four more times for refolding of the tetradecamer complex, before concentrating and buffer exchanging in 200 mM ammonium acetate and diluting to a working solution of 2.5 µM.

A 10 µM stock of β-galactosidase (by tetramer mass) solution was made by dissolving the lyophilized powder in 150 mM ammonium acetate buffer. The solution was then concentrated and diluted 5 times using a 10 kDa molecular weight-cutoff filter using the buffer mentioned above. The working solution was prepared by diluting the stock to a β-galactosidase concentration of 2.5 µM.

*E. coli* ribosome, purchased at a stock concentration of 13 µM, was buffer exchanged 5 times using a 10 kDa molecular weight-cutoff filter into a buffer containing 150 mM ammonium acetate, 10 mM magnesium acetate, and 25 mM triethylammonium acetate at pH 7.4. The sample was diluted to a working solution of 0.5 µM ribosome using a buffer containing 150 mM ammonium acetate and 0.5 mM magnesium acetate at pH 7.4.

### Reagent preparation

PFDA comes as a viscous liquid, so the stock vial was placed in a warm water bath (∼40 °C) for about 15 min to allow it to be drawn in a pipette. The volume of PFDA was weighed to be 1.22 mg and dissolved in methanol to 2.16 mM. The stock solution was then diluted to 216 µM with 10% methanol and 2% glacial acetic acid and served as the working solution.

A 1 mg mL^−1^ stock solution of proton sponge in water was made and then diluted to 701 µM in 19% methanol and 1% glacial acetic acid to make the working solution.

Ubiquitin used as the multiply-charged ion attachment (MIA) reagent for 30S ribosome was prepared by diluting the stock to 2.9 µM in 2% glacial acetic acid and 8% acetonitrile. Working solution of ubiquitin for MIA of GroEL was prepared by diluting the stock to 10 µM in 20% methanol and 3% glacial acetic acid.

### Mass spectrometry

All experiments were performed on a Sciex TripleTOF 5600 hybrid quadrupole/time-of-flight (QqTOF) tandem mass spectrometer (Sciex, Concord, ON, Canada) which was previously modified to enable the mutual storage of ions of opposite charges and ion/ion reactions.^74^ All reactions were tuned to maximize signal of the analyte and injection efficiency of the reagent to achieve pseudo-first order kinetics. Briefly, analyte anions (A_30_, GroEL) were generated *via* nano-ESI (nESI). For analyte anions with *m*/*z* values below 3100 (*e.g.*, A_30_ and ubiquitin), mass-selection was performed using Q1 followed by accumulation in q2 (inscribed radius = 4.17 mm) operated at a drive frequency of 1.843 MHz. For A_30_ specifically, a broad isolation of charge states 18^−^ to 13^−^ was required to avoid boundary activation of the highly charged intact analyte. Analyte anions with *m*/*z* values above 3100 are too high in *m*/*z* for Q1 to isolate. Instead, the ions were trapped in q2 without mass selection. Unwanted ions were removed in two steps: first, dipolar DC (DDC) was applied to eject higher-*m*/*z* ions, and then a stored waveform inverse Fourier transform (SWIFT) isolation waveform (generated externally using an Agilent 33220B source) was applied to remove lower-*m*/*z* ions. This sequence produced a purified population of desired high-*m*/*z* analyte anions for subsequent ion/ion reactions. PFDA (*m*/*z* 464) and proton sponge (*m*/*z* 215) cations were generated *via* nano-ESI (injection times ranging from 200–425 ms), mass-selected *via* Q1, and transmitted through q2. Ion/ion reactions were performed in transmission mode^75^ for PFDA or proton sponge through q2 with potentials on the end caps to trap analyte anions. Product anions were then sent to the orthogonal TOF for mass analysis. Calibration of the instrument was done *via* a two-step process previously described^74^ using CsI and β-galactosidase.

The proton transfer reactions involving anions of ubiquitin were performed in trapping mode. Ubiquitin anions ([U − 5H]^5−^) were mass selected in Q1 and stored in q2. For the introduction of PFDA and PS cations, first they were mass selected in Q1 then trapped in q2 (at *q*_u_ values 0.784 and 0.806, respectively) by applying 100 kHz AC voltages to the end caps. During injection of reagent cations the entrance and exit end caps were set to 50 and 100 V_0−p_, respectively. During mutual storage, both end caps were set to 100 V_0−p_. Mutual storage times of 100–200 ms were used. We note that the tendency for reagent ion attachment has not been observed to differ whether using trapping or transmission mode ion/ion reactions.

The nPTRs of *E. coli* β-galactosidase and 30S ribosome were also done in trapping mode. Isolations of β-galactosidase and 30S ribosome were done as previously described for *m*/*z* greater than 3100. PFDA was injected *via* Q1 mass selection into q2 (at *q*_u_ values 0.784 and 0.853, respectively) with 140–150 and 290 V_0−p_ at 100 kHz for the entrance and exit end caps, respectively. For mutual storage both end caps were held at 290 V_0−p_ for 16–125 ms.

## Results and discussion

Criteria for a proton transfer reagent ion for high mass analyte ions in an ion trap mutual storage experiment, in either polarity, include: (1) ready availability at low cost, (2) high ionization yield *via* an available ionization method, (3) minimal fragmentation arising from the ion/ion reaction, (4) minimal ion/ion attachment, and (5) the ability to store the reagent ion simultaneously with high *m*/*z* analyte product ions. Criterion 1 is an obvious practical consideration. Criterion 2 leads to minimal ion fill times for the reagent as well as high reaction rates. In essence, the reagent ion should be cheap and easy to make in high abundance. The reagent emphasized here, PFDA, is commercially available at moderate cost and can be readily generated as [PFDA + H^+^] both *via* ESI and APCI, thereby satisfying criteria 1 and 2.

Criterion 3 relates to the likelihood for fragmentation as a result of the ion/ion reaction. Analyte ion fragmentation might be desirable if the ion/ion reaction is intended to be a dissociation method, as with electron transfer dissociation,^76^ but is undesirable if the purpose of the reaction is simply to reduce the charge state. The likelihood for fragmentation of an ion/ion reaction analyte product ion is related to the reaction exothermicity, how the reaction exothermicity is partitioned, product ion kinetic stability, the pressure and temperature of the bath gas in the reaction region, and the number of degrees of freedom of the analyte.^77^ All gas-phase ion/ion mutual neutralization reactions, either full or partial, are highly exothermic. Under common ion trap operating conditions with a bath gas pressure of 1–10 mTorr, fragmentation resulting from the removal of protons from multiply-charged cations has not been noted. On the other hand, the protonation of multiply-charged anions by a cation has been known to lead to fragmentation, at least for relatively small and labile anions.^64,77^ However, the high numbers of degrees of freedom associated with high mass polyatomic ions as well as the high collisional cooling rates at 1–10 mTorr^78,79^ minimizes the likelihood of fragmentation *via* proton transfer to a multiply-charged high mass anion.

**Fig. 1:**
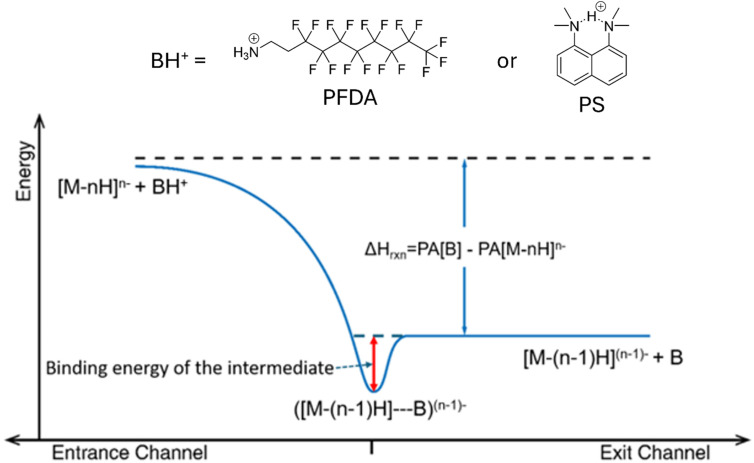
Hypothetical energy diagram for proton transfer to a multiply-deprotonated analyte, [M − *n*H]^*n*−^, from a protonated base, BH^+^ (PFDA or PS).

A gas-phase ion/ion reaction can take place *via* the transfer of a proton at a crossing point on the energy hypersurface, referred to as a proton ‘hopping’ mechanism, or *via* a long-lived proton-bound intermediate.^80^ The hopping mechanism does not require the formation of a long-lived complex and can occur *via* a ‘fly-by’ interaction. However, as the physical cross-sections of the two ions increase, the likelihood of a collision leading to a long-lived complex increases. If the binding interaction between the two collision partners is high, the complex can be collisionally stabilized leading to the attachment of the reagent ion to the analyte ion.^81^Fig. 1 shows a hypothetical energy diagram with the entrance channel (*i.e.*, the two reactants) shown on the left and the exit channel (*i.e.*, the two products) shown on the right. The intermediate is shown in the middle with a red arrow showing the binding energy of the intermediate with respect to the products. From the standpoint of charge and mass determination, either exclusive proton transfer or exclusive ion attachment is preferred. A combination of both mechanisms can complicate spectral interpretation. Hence, the extent to which ion attachment occurs, criterion 4, and how readily the adduct can be removed, if needed, is an important figure of merit for a proton transfer reagent.

In this work, we compare protonated PFDA to protonated 1,8-bis(dimethylamino)naphthalene (proton sponge, 214.3 Da), a reagent used in previous negative ion charge reduction experiments.^77^Table 1 lists the calculated binding energies (red arrow in Fig. 1) associated with the proton transfer intermediates for the reaction of protonated PFDA or protonated proton sponge with the model glycine C-terminal carboxylate or dimethyl phosphate sites (see SI for details). The proton transfer intermediates involve the interactions between neutral PFDA or proton sponge and the amino acid or dimethyl phosphoric acid. Binding energies for the phosphate site are greater for each cation than the carboxylate site and protonated proton sponge shows greater binding for both anion sites. The values in the Table 1, therefore, lead to the expectation that protonated PFDA will show a lesser tendency for ion attachment to negatively-charged analyte ions than protonated proton sponge. (*i.e.*, PFDA shows a lesser tendency to attach to polypeptide analyte anions, for which carboxylate groups are the favored anionic sites, relative to nucleic acid analytes, for which phosphate groups are the preferred anionic sites.)

**Table 1:** Calculated binding energies associated with B-HA where B = proton sponge or PFDA and HA = the glycine C-terminus or the phosphoric acid dimethyl ester. Calculation details provided in SI along with the lowest energy simulated structure of electrostatically bound complex of protonated proton sponge and deprotonated dimethyl phosphate (Fig. S1)

	Binding energy (kcal mol^−1^)	
	Proton sponge	PFDA
Glycine C-terminus	24.06	14.92
Phosphoric acid dimethyl ester	60.91	20.34

**Fig. 2:**
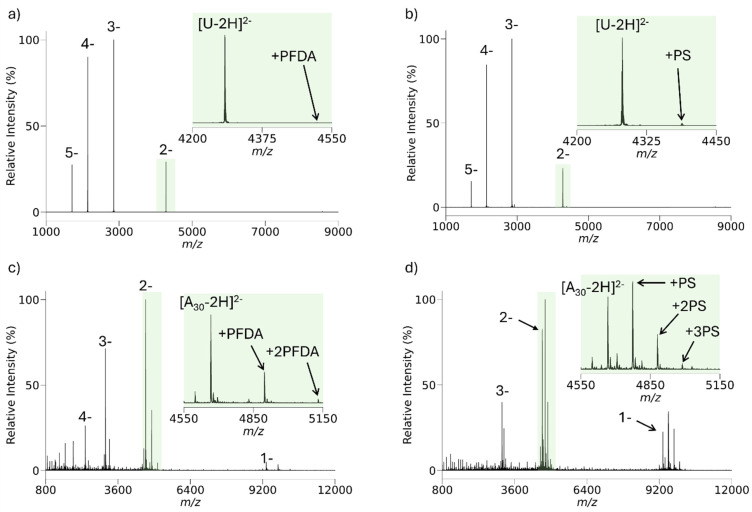
(a) Post-ion/ion reaction spectrum of [U − 5H]^5−^/protonated PFDA. (b) Post-ion/ion reaction spectrum of [U − 5H]^5−^/protonated proton sponge. (c) Post-ion/ion reaction spectrum of [A_30_–(18 − 13)H]^(18−13)−^/protonated PFDA. (d) Post-ion/ion reaction spectrum of [A_30_–(18 – 13)H]^(18−13)−^/proton sponge.

Fig. 2 compares results for an analyte with carboxylate charge bearing sites, anions of ubiquitin, to those for an analyte with phosphate charge bearing sites, polyadenylate 30-mer anions, in reactions with protonated proton sponge and protonated PFDA. (Spectra showing the reactant ions after isolation and prior to ion/ion reaction are provided in Fig. S2.) The extent of cation attachment to the analyte ions is dependent upon both the identities of the reagent cations and the identities of the charge bearing sites in the analyte anions. Consistent with Table 1, the [M − 5H]^5−^ anion of ubiquitin showed little attachment with either reagent cation. While both signals were small, the proton sponge showed more attachment than protonated PFDA. In the case of the oligonucleotide anions, a broad analyte ion isolation step that included the 18^−^ to 13^−^ charge states of 5′-(A_30_)-3′ (see the Fig. S2b) resulted in significant degrees of cation attachment for both reagent cations. However, the extent of attachment was markedly greater for the proton sponge. As expected, the likelihood for reagent cation attachment is greater for the phosphate containing analyte anions and for the proton sponge, consistent with Table 1.

**Fig. 3:**
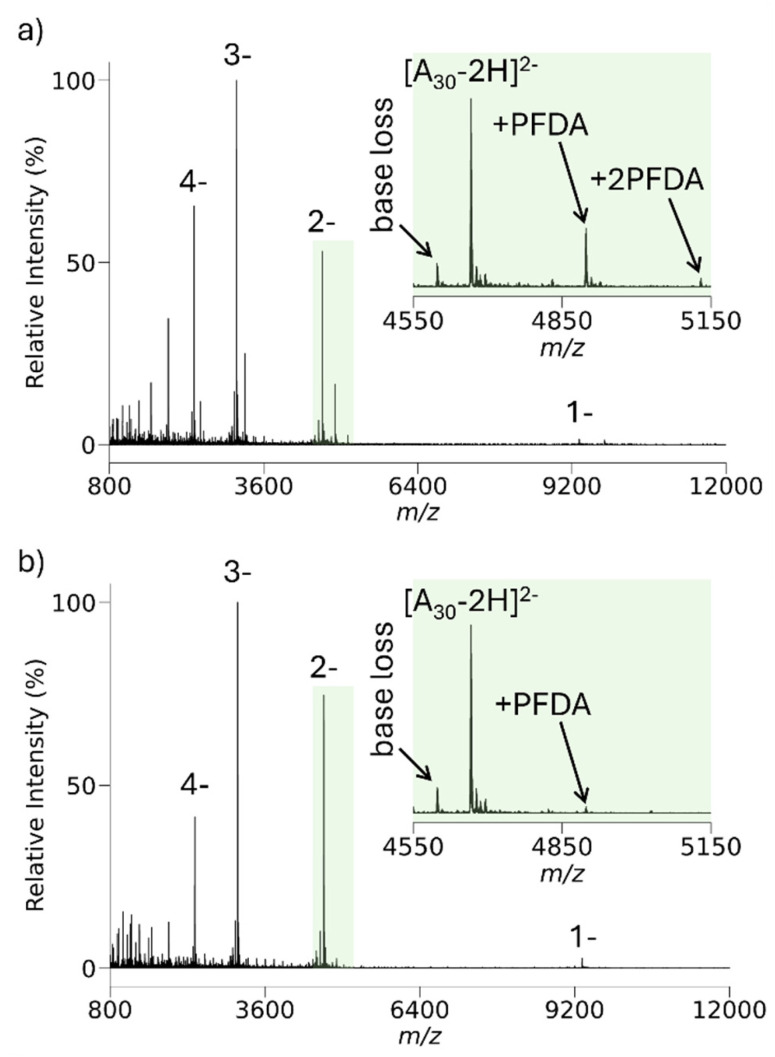
(a) Negative mode nESI mass spectrum of 5′-d(A)_30_-3′ after ion/ion proton transfer with protonated PFDA. (b) Post-ion/ion reaction product ion spectrum after application of 50 V DDC at a low-*m*/*z* cut-off of 450 (*V*_RF_ = 2469 V_0−p_).

Ion attachment need not be problematic if it is possible to dissociate the weakly-bound complexes prior to mass analysis. Fig. 3 summarizes an ion/ion reaction experiment very similar to the experiment leading to Fig. 2c with an additional 100 ms period of DDC collisional activation. DDC is a broad-band collisional activation technique that is useful for heating ions on a non-resonance basis in an electrodynamic ion trap.^82–85^Fig. 3a shows the post-reaction products of A_30_ and PFDA. Note that there is evidence for the loss of a single adenine nucleobase, which likely arises from collisional activation in the process of transmitting and trapping the analyte ions. Loss of adenine, either as an ion from relatively high charge states or as a neutral at relatively low charge states, is known to be a particularly facile process for multiply-deprotonated oligodeoxynucleotides.^86,87^ The application of a 50 V DDC at a low-mass cut-off of *m*/*z* 450 to the ions of Fig. 3a results in the spectrum shown in Fig. 3b. Using a modified form of the Tolmachev model for estimating the change in the effective temperature of ions subjected to the DDC conditions used here yields a temperature increase of roughly 64 K. This is sufficient to dissociate the PFDA adducts without resulting in significant cleavage of covalent bonds, at least for the 3^−^ and 2^−^ charge states. This experiment suggests that the PFDA reagent can be useful in manipulating the charge states of nucleic acid anions, provided means for supplemental heating are available.

**Fig. 4:**
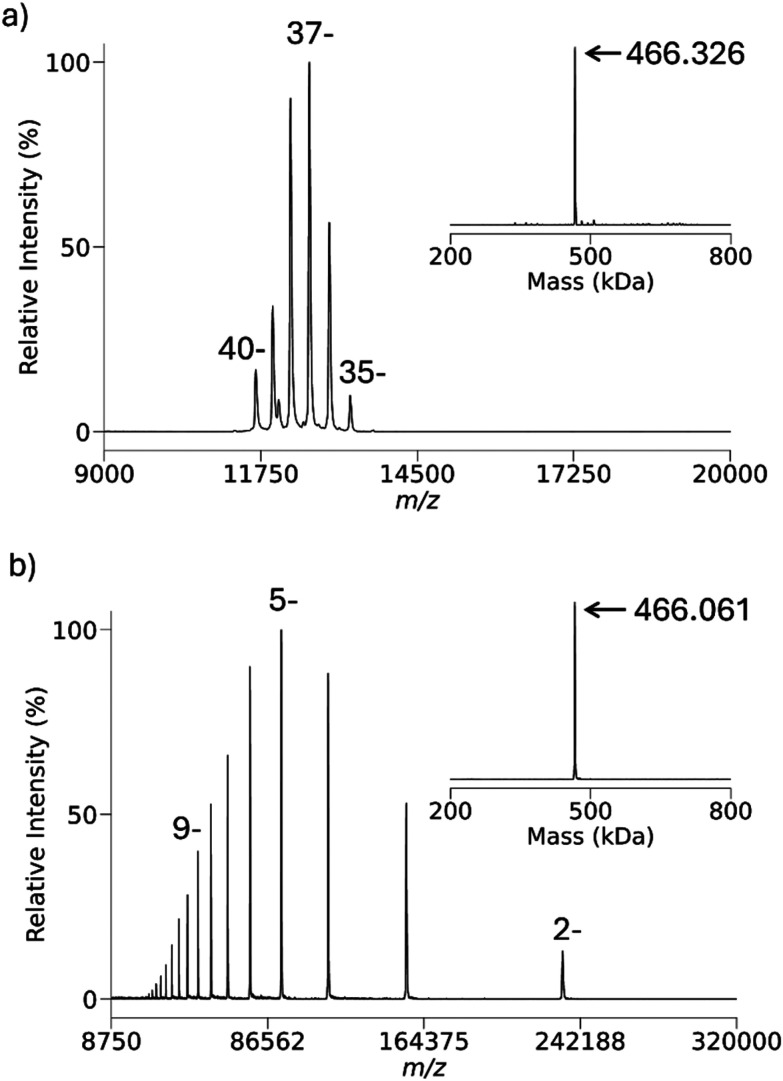
(a) Negative ion nESI of β-galactosidase. A zero-charge deconvolution of the pre-ion/ion reaction charge states is shown in the inset. (b) Post-ion/ion reaction spectrum after reaction with protonated PFDA. The zero-charge deconvolution of the post-ion/ion reaction spectrum is shown in the inset in the bottom spectrum.

The number of degrees of freedom tends to increase the lifetime of a complex. It is of interest, therefore, to determine the extent to which ion attachment of protonated PFDA might occur for much larger analytes than ubiquitin. Fig. 4 summarizes the ion/ion reaction experiment involving the reactions of the 37^−^ to 40^−^ charge states of *E. coli* β-galactosidase with protonated PFDA. Fig. 4a show the isolated precursor analyte charge states while the inset on the right shows the results of a UniDec zero-charge deconvolution^88^ of the precursor analyte charge states (all UniDec parameters are shown in Table S3). Fig. 4b shows the post-ion/ion reaction spectrum with the inset showing the zero-charge UniDec deconvolution applied to the β-galactosidase ions after charge reduction. The difference between the two deconvolution results is significantly lower than the mass of a single PFDA molecule and falls within the error of the measurement, thereby suggesting essentially no ion attachment. The analogous experiment with GroEL (see below) also shows no evidence for ion attachment. The experiments with ubiquitin, β-galactosidase, and GroEL anions, therefore indicate that protonated PFDA can be used to protonate anions with carboxylate charge-bearing sites with minimal to no ion attachment.

**Fig. 5:**
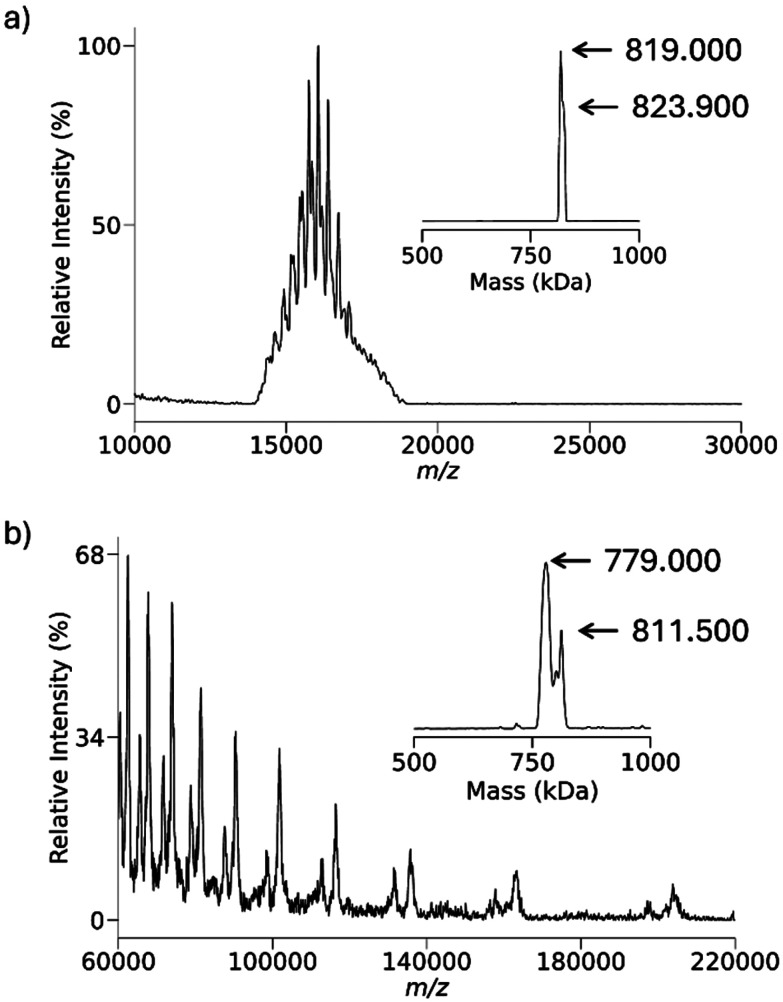
(a) Isolated charge states of anions derived from negative nESI of *E. coli* 30S ribosome particles. The inset shows the results of the UniDec zero-charge deconvolution of the ions in (a). (b) Post-ion/ion reactions spectrum following reactions of protonated PFDA with the anions of (a). The inset is the result of the UniDec zero-charge deconvolution of the product ions of (b).

The 30S *E. coli* ribosome particle, a nucleoprotein complex comprised of 16S ribosomal RNA (1542 nucleotides) and roughly twenty proteins, was chosen to generate multiply-charged anions in which many or most of the charge-bearing sites are likely to be phosphates. Fig. 5a shows the isolated region of 30S precursor charge states and Fig. 5b shows a post-ion/ion reaction spectrum following reactions with protonated PFDA. The inset in Fig. 5a shows the results of the UniDec zero-charge deconvolution of the precursor ion population. It is clear that there is a mixture of charge states in the precursor ion population, which suggests a mixture of components. Given the limited number of poorly resolved charge states, the deconvolution does not show clearly resolved components. The inset to Fig. 5b shows the UniDec zero-charge deconvolution of the post-ion/ion reaction product ions and shows two major components at nominally 779 kDa and 811.5 kDa, which both differ from the deconvolution results for the precursor ions. Given the possibility for the adduction of PFDA cations to the complexes, we determined the masses of the major particles in the 30S mixture using the attachment of the 7^+^ charge state of ubiquitin. The multiply-charged ion attachment (MIA) approach relies on the exclusive attachment of the reagent ions for charge state reduction. We have previously reported the use of MIA to *E. coli* ribosome cations^89^ and anions^90^ for the determination of their masses. The MIA experiments performed here are summarized in Fig. S3 and Table S1 with the results indicating that the major components correspond to the 30S particle missing the S1 protein (30S–S1, 800 kDa) and the 30S particle missing both the S1 and S2 proteins (30S–S1–S2, 773 kDa). These components were also observed in our previous MIA work with 30S anions.^92^

**Fig. 6:**
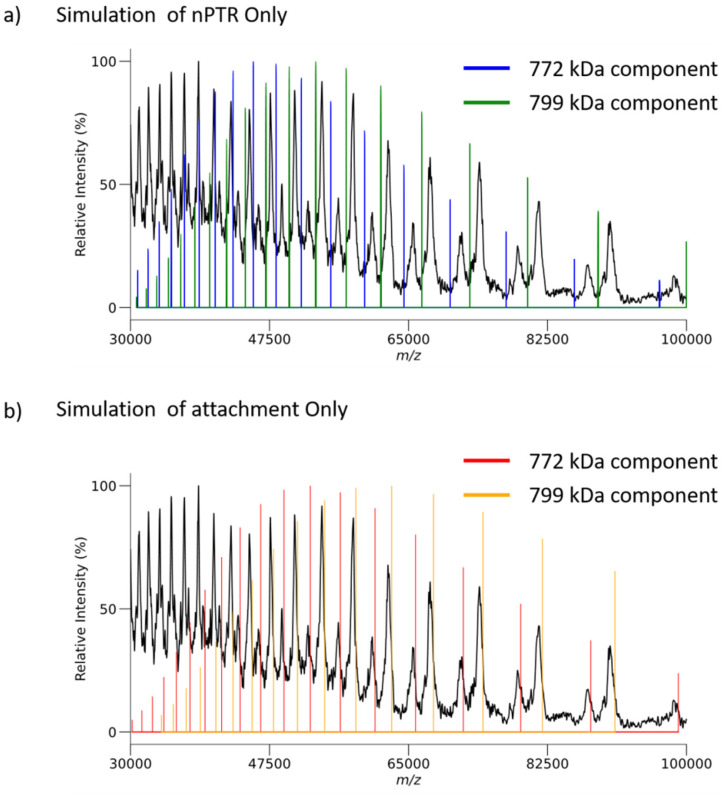
(a) Comparison of expected locations for the 30S–S1–S2 charge states (772 kDa, blue lines) and 30S–S1 (799 kDa, green lines) resulting from exclusive proton transfer *versus* experimental data. (b) Comparison of expected locations for the 30S–S1–S2 charge states (772 kDa, red lines) and 30S–S1 (799 kDa, orange lines) resulting from exclusive attachment *versus* experimental data.

Fig. 6a compares the locations on the *m*/*z*-scale of the expected charge states for 30S–S1–S2 (772 kDa, blue vertical lines) and 30S–S1 (799 kDa, green vertical lines) product ions that would be generated exclusively *via* proton transfer (*i.e.*, no attachment) with the experimental data. The experimental results consistently show the product ions to significantly exceed the expected *m*/*z* values for proton transfer with no ion attachment. Fig. 6b compares the locations on the *m*/*z*-scale of the expected charge state for the 30S–S1–S2 (772 kDa, red vertical lines) and 30S–S1 (799 kDa, orange vertical lines) product ions generated exclusively *via* attachment with the experimental data. The experimental results consistently fall at lower *m*/*z* values than expected on the basis of exclusive attachment. In comparing Fig. 6a with Fig. 6b, it is apparent that there is more ion attachment than proton transfer. The ribosome 30S results demonstrate that, unlike with deprotonated proteins, ion attachment competes with proton transfer in reactions with nucleic acid anions. The supplemental heating employed to drive off unwanted adduction in Fig. 3b is not possible in exceptionally high *m*/*z* products due to the high *m*/*z* cutoff seen in DDC activation. For example, heating these product ions to the same temperature as before results in a cutoff above *m*/*z* 19 600. Resonant activation, *via* a targeted frequency or SWIFT, is also not feasible for ions bearing secular frequencies below 5 kHz due to high pass filters prior to signal transmission to the rods themselves.

Criterion 5 relates to the *m*/*z* range over which analyte and reagent ions can be mutually stored within an electrodynamic trap. The low *m*/*z* limit is determined by the low *m*/*z*-cutoff (LMCO), as given for a linear ion trap by:1
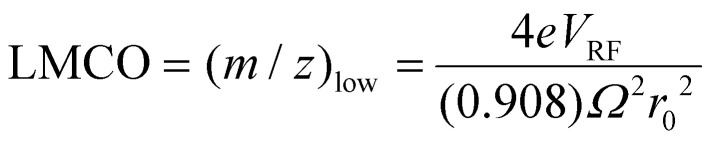
where *V*_RF_ is the 0–p maximum amplitude of the RF voltage applied to the quadrupole rods, *Ω* is the frequency of the RF voltage, *r*_0_ is the inscribed radius of the rod array, and *e* is the elementary charge. The upper *m*/*z* limit is not precisely defined but is ultimately limited by the so-called Dehmelt pseudo-potential well-depth approximation,^91,92^*D*_u_, which is the effective trapping well experienced by an ion in the trap in dimension *u* (*viz*., *x* or *y* in a linear ion trap). The Dehmelt pseudo-potential well-depth is valid for *q*_u_ values less than 0.4, which is the case forall of the ions discussed here. When the ion kinetic energy approaches *zeD*_u_, the trapping potential energy, ions can ‘evaporate’ from the ion trap. For thermalized ions, ion cloud radii approach *r*_0_ when the kinetic energies of the trapped ions approach *zeD*_u_. For a linear ion trap,2
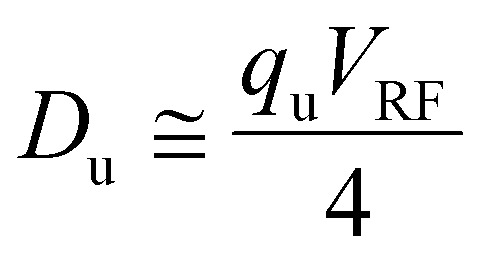
where the dimensionless Mathieu parameter, *q*_u_, for the RF component of the quadrupolar potential is given by:3
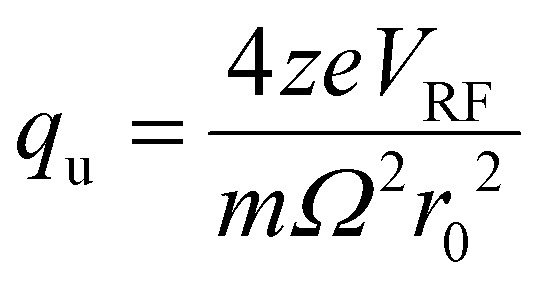


The approximate root mean square average radius of an ion cloud at temperature *T* is given by:^93^4
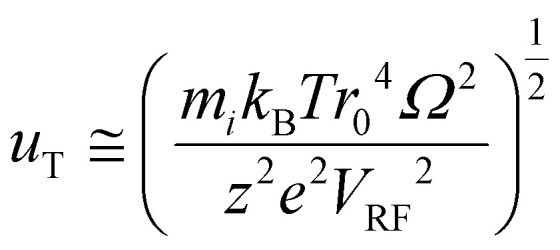


Hence, both the low and high *m*/*z* limits increase with *V*_RF_. For this reason, a high *m*/*z* reagent ion is highly desirable when the analyte ion is of high *m*/*z*.

**Fig. 7:**
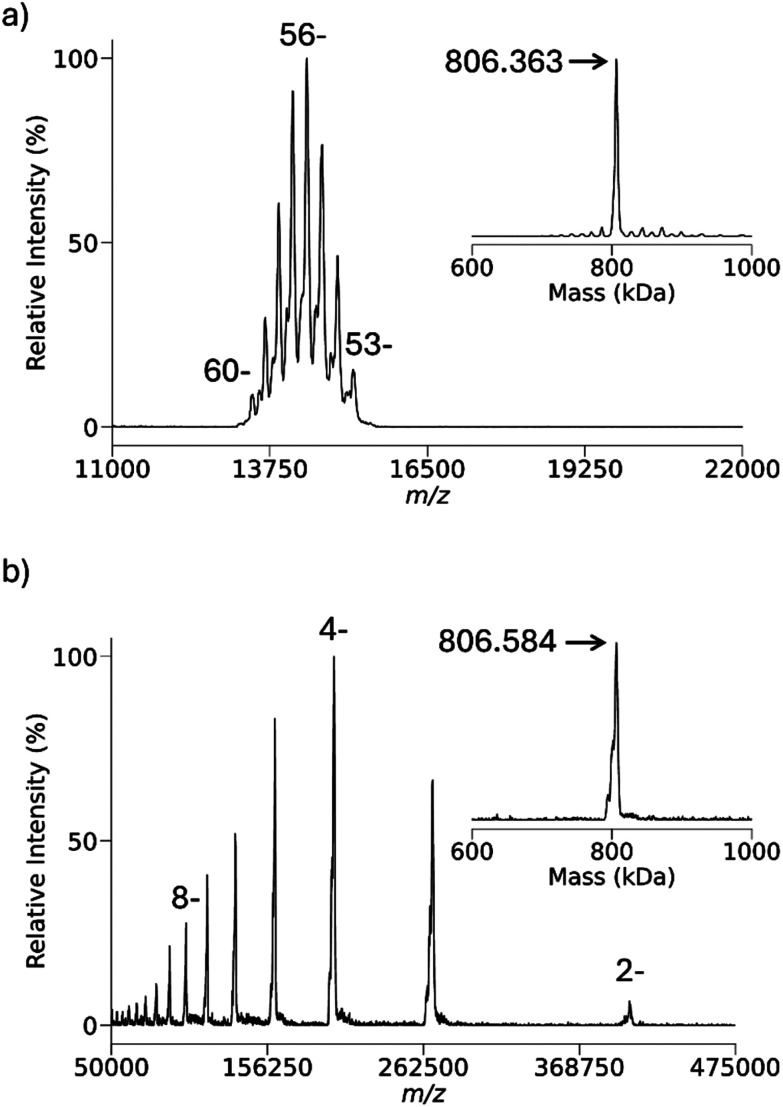
(a) Pre-ion/ion charge states of isolated *E. coli* GroEL anions with the inset showing the UniDec zero-charge deconvolution. (b) Spectrum of post-ion/ion reaction with protonated PFDA with the inset showing the UniDec zero-charge deconvolution.

Fig. 7 illustrates the *m*/*z* range accessible using protonated PFDA with anions derived from *E. coli* GroEL as the analyte using the present apparatus. A precursor analyte ion population of the 53^−^ to 60^−^ charge states were subjected to reaction with protonated PFDA using a low *m*/*z* cutoff of *m*/*z* 420 (*V*_RF_ = 2304 V_0−p_). Product ions were generated down to the 2^−^ ion. The UniDec deconvolution of the GroEL charge states both before and after reaction yields essentially the same mass (806 kDa). A MIA experiment with the 8^+^ charge state of bovine ubiquitin was performed to evaluate the extent of possible adduction in the protonated PFDA experiment of Fig. 7, as summarized in Fig. S4 and Table S2. The MIA experiment also yielded a mass of 806 kDa, which reinforces the conclusion that ion attachment of PFDA is minimal to absent with protein complexes in which the negative charge is localized at carboxylate sites. The ion cloud radius of the 2^−^ ions is approximately 0.64 mm with a *zeD* = 1.1 eV. The radius of the exit aperture from the linear ion trap used to store the ions^74^ is 2 mm while the slit width for injection into the time-of-flight (TOF) pulse-out region is also 2 mm. Hence ion cloud radii of 1 mm or greater cannot fully pass through the slit to the TOF. Under the same conditions, 1^−^ ions, which were not observed, would have an estimated radius of 1.3 mm. The detection efficiency of the channelplate detector in this system also decreases significantly with charge state for ions of this size.^74^ Hence, the combination of increasing product ion cloud size along with decreasing charge combine to limit performance at the high end of the *m*/*z* range.

## Conclusions

There are several criteria associated with the utility of a reagent cation for the charge-state manipulation of analyte anions *via* proton transfer in an electrodynamic ion trap. Practical considerations include the cost and availability of the reagent and the ease with which the protonated form can be generated *via* an available ionization method. The reagent emphasized here, PFDA, is commercially available and is readily ionized *via* both spray ionization and atmospheric pressure chemical ionization. Many commercial instruments are outfitted with an APCI reagent source, so protonated PFDA can be readily generated on such platforms. Criteria relating to the chemistry of the proton transfer reaction include the propensity for analyte fragmentation and the tendency for ion attachment. The fragmentation tendency is dependent upon the exothermicity of the reaction, the kinetic stability of the product ion, the number of degrees of freedom, and the cooling rate in the ion trap. For high mass analyte ions, fragmentation tends to be minimized due to the high numbers of degrees of freedom and relatively large collision cross-sections of the analyte, which lead to long lifetimes and high collisional cooling rates, respectively. The tendency for attachment depends on the interaction strengths between the reagent and analyte reaction sites as well as the cooling rate. The primary amine of PFDA is a relatively weakly interacting site with carboxylic acids and somewhat more strongly interacting site with phosphoric acids. Hence, the tendency for attachment is lower for protein analyte anions than oligonucleotide anions. This is largely a problem for unresolved charge-state oligonucleotide species due to the ambiguity in the number of attachments. In either case, mild heating gives rise to loss of the reagent adduct as a neutral species. Other activation methods such as ion-trap collision induced dissociation (CID), beam-type CID, and infrared-multiphoton dissociation (IRMPD) would be feasible alternatives to DDC for adduct removal. Specifically, IRMPD would be the best option for high *m*/*z* analytes since it does not require ion acceleration in a shallow well-depth. The final criterion, which is specific to ion traps, relates to mass of the reagent whereby higher masses allow for mutual storage with high *m*/*z* analyte ions. At *m*/*z* 464, protonated PFDA allows for the use of storage conditions that allow for mutual storage of analyte ions in excess of *m*/*z* 300 000 shown here using a linear ion trap collision cell of a commercial quadrupole/time-of-flight tandem mass spectrometer. Charge reduction *via* protonated PFDA reactions coupled with activation with a broadband technique, such as IRMPD, would enable more facile top-down analysis of high *m*/*z* analyte anions.

## Author contributions

Nicholas R. Ellin: conception, investigation, data curation, methodology, visualization, writing – original draft preparation. Boukar K. S. Faye: conception, investigation, data curation, visualization. Seth A. Horn: conception, investigation, data curation, visualization. Alex M. Koers: conception, investigation, data curation, visualization. Scott A. McLuckey: methodology, resources, supervision, visualization, writing – review and editing.

## Conflicts of interest

The authors declare no competing financial interests.

## Supplementary Material

AN-151-D5AN01354B-s001

## Data Availability

The data that support the findings of this study are available from the corresponding author upon reasonable request. Supplementary information (SI): Details about the calculations associated with Table 1 are provided. Ion isolation spectra related to Fig. 2 are provided along with the results from multiply-charged ion attachment to anions of *E. coli* ribosome 30S particles and GroEL. A table summarizing the UniDec parameters used for the pre- and post-ion/ion reaction data is included. See DOI: https://doi.org/10.1039/d5an01354b.
