# Supplementary material for: Proton transfer reagent cations for ion–ion charge state manipulation of high mass negatively-charged analytes in an electrodynamic ion trap
Source: Analyst. 2026 Feb 18;151(7):1902–13. doi: 10.1039/d5an01354b (PMC12935111; doi:10.1039/d5an01354b)
Supplement: AN-151-D5AN01354B-s001 [file AN-151-D5AN01354B-s001.pdf]

Supplementary Information (SI) for Analyst.
This journal is © The Royal Society of Chemistry 2026

Supporting Information for

Proton Transfer Reagent Cations for Ion-Ion Charge State Manipulation of High Mass Negatively-charged Analytes in an Electrodynamic Ion Trap

Nicholas R. Ellin†, Boukar K.S. Faye†, Seth A. Horn, Alexander M. Koers, Scott A. McLuckey*

Department of Chemistry
Purdue University
West Lafayette, IN 47907-2084

† Authors contributed equally
*Address correspondence to:

Dr. Scott A. McLuckey
560 Oval Drive
Department of Chemistry
Purdue University
West Lafayette, IN 47907-2084, USA
Phone: (765) 494-5270
Fax: (765) 494-0239
E-mail: mcluckey@purdue.edu

Table of Contents

Summary of calculations	S3
Figure S1	S9
Figure S2	S10
Figure S3	S11
Figure S4	S12
Table S1	S11
Table S2	S12
Table S3	S13

Calculation Details:

Values for binding energies communicated in **Table 1** were determined via calculation of the change in electronic energies between bound and unbound states of Proton Sponge and PFDA with a C-terminal glycine residue and phosphoric acid dimethyl ester (red arrow in **Figure 1**). C-terminal glycine residue and phosphoric acid dimethyl ester are utilized as model systems for polypeptide and nucleic acid analytes. Dimer noncovalent binding energies were calculated as the difference between the electronic energy of the optimized dimers and the sum of the optimized monomer energies at the rB3LYP/6-311+G(D,P) level, using an ultrafine grid and tight SCF convergence. All calculations were performed in the gas phase with Gaussian 16.

Coordinates for calculated structures

Non-complexed Species

Dimethyl phosphoric acid

P	-0.00329100	0.36630000	0.10573100
O	0.04868100	0.55023500	1.57190000
O	-1.43662900	0.09774900	-0.54029500
O	0.84899400	-0.85390300	-0.48148200
O	0.52419800	1.62217300	-0.75326600
H	0.42644800	2.45151200	-0.27063700
C	-2.26525900	-0.96121000	-0.01743400
H	-3.22771300	-0.86213400	-0.51404200
H	-1.82244600	-1.93087600	-0.25305400
H	-2.38517300	-0.85142100	1.06178200
C	2.19742200	-1.07409200	-0.02056100
H	2.51235900	-2.02186500	-0.45133900
H	2.84894700	-0.27233200	-0.37463400
H	2.22201300	-1.12561000	1.06907000

Glycine

C	0.53768500	0.10134600	0.01651700
O	0.59198000	1.31204200	0.05191200
O	1.63680400	-0.68267800	-0.08106000
H	2.41044400	-0.09379500	-0.09673800
C	-0.72952900	-0.71596500	0.12732500
H	-0.65171300	-1.57461900	-0.54774600
H	-0.72516900	-1.12248000	1.15508700
N	-1.88517900	0.10047900	-0.20839300
H	-2.74181500	-0.30042900	0.15684100
H	-1.77470400	1.04077600	0.16144200

PFDA

N	-0.65054900	7.81058700	0.00000000
H	-1.24523400	7.85286400	0.82052300
C	0.19414200	6.62277300	0.00000000
H	0.84364700	6.66452800	-0.87643800
H	0.84364700	6.66452800	0.87643800
C	-0.58182200	5.28806500	0.00000000
H	-1.22232600	5.22556400	-0.88278000
H	-1.22232600	5.22556400	0.88278000
C	0.33584500	4.08194600	0.00000000
F	1.14958700	4.10223100	1.09876900
F	1.14958700	4.10223100	-1.09876900
H	-1.24523400	7.85286400	-0.82052300
C	-0.44523400	2.72914600	0.00000000
C	0.42326900	1.42416900	0.00000000
F	-1.24182900	2.71710800	-1.09889700
F	-1.24182900	2.71710800	1.09889700
F	1.20741800	1.43311300	-1.09858200
F	1.20741800	1.43311300	1.09858200
C	-0.41008900	0.08924600	0.00000000
C	0.46880500	-1.21616900	0.00000000
C	-0.36515300	-2.54903900	0.00000000
C	0.50514800	-3.85279800	0.00000000
C	-0.31658300	-5.18581300	0.00000000
F	-1.19509200	0.07137900	1.09805200
F	-1.19509200	0.07137900	-1.09805200
F	1.25254200	-1.20977700	1.09816500
F	1.25254200	-1.20977700	-1.09816500
F	-1.15039000	-2.57272900	-1.09811400
F	-1.15039000	-2.57272900	1.09811400
F	1.28870200	-3.86856900	-1.09813500
F	1.28870200	-3.86856900	1.09813500
F	-1.08639500	-5.27696800	-1.08727900
F	-1.08639500	-5.27696800	1.08727900
F	0.54554900	-6.20815100	0.00000000

Proton Sponge

C	-2.44423500	0.10161100	-0.01589000
C	-1.28391700	0.86066600	0.00461500
C	0.00000000	0.20606800	0.02234300
C	0.00000100	-1.23697500	0.02970100
C	-1.21941000	-1.95968600	0.01631000
C	-2.41923500	-1.30020900	-0.01495100
H	-3.39936100	0.61682400	-0.03013400

C	1.28391500	0.86066900	0.00460600
C	1.21941300	-1.95968500	0.01632600
H	-1.17801900	-3.04235300	0.02140000
H	-3.35280800	-1.85070900	-0.03007000
C	2.41923700	-1.30020500	-0.01492500
C	2.44423500	0.10161400	-0.01587900
H	1.17802400	-3.04235100	0.02141900
H	3.35281100	-1.85070400	-0.03003200
H	3.39936100	0.61682800	-0.03012800
N	1.43683200	2.25692300	-0.08374800
H	0.87844300	2.81889900	0.53639200
H	2.40518400	2.53728100	-0.00841500
N	-1.43683400	2.25692500	-0.08369300
H	-2.40518800	2.53727500	-0.00837500
H	-0.87847000	2.81887000	0.53649900

Electrostatically Bound Complexes

Glycine and PFDA

N	6.14119700	-1.50342200	-0.75855600
H	6.25421600	-0.79005900	-1.47884100
C	4.99606600	-1.18459900	0.10322400
H	4.92234400	-1.95671700	0.87362600
H	5.21311000	-0.23820700	0.60424500
C	3.66729800	-1.07340900	-0.66483700
H	3.46942500	-1.99418500	-1.22121400
H	3.71747600	-0.24942700	-1.38386500
C	2.48869700	-0.82562400	0.25829100
F	2.70838700	0.28670700	1.02432400
F	2.32549200	-1.88357800	1.11950000
H	6.03521500	-2.41009000	-1.20710500
C	1.14356100	-0.62016400	-0.50172100
C	-0.14863300	-0.61945000	0.38609100
F	1.01410200	-1.62345200	-1.41359100
F	1.22953100	0.55509600	-1.18034700
F	-0.41523300	-1.90252800	0.72489400
F	0.07905800	0.08794300	1.51765900
C	-1.39879700	0.01143600	-0.32526300
C	-2.76591900	-0.24735500	0.39533300
C	-4.01435600	0.34981000	-0.34805400
C	-5.30157900	0.40963000	0.54261900
C	-6.63061400	0.60997900	-0.25382100
F	-1.19711300	1.34733500	-0.39198000
F	-1.48925700	-0.48719900	-1.58463000
F	-2.69712800	0.29090700	1.63803300
F	-2.96624900	-1.58056200	0.50253600

F	-4.26811500	-0.42505700	-1.43120100
F	-3.74957600	1.60673300	-0.77134200
F	-5.42472700	-0.73924100	1.24987200
F	-5.18141700	1.44493900	1.40421400
F	-6.93793600	-0.48803000	-0.95613600
F	-6.52927500	1.65257000	-1.09239700
F	-7.62129100	0.85137700	0.61695300
C	8.34993700	0.69135200	-0.03370100
O	7.52487300	1.11018800	-0.83665900
O	8.35171700	-0.54892400	0.45097700
H	7.57524800	-1.05565300	0.03780000
C	9.50744200	1.52545200	0.47595700
H	9.66013600	1.29386600	1.53529300
H	10.39747800	1.14819400	-0.05919500
N	9.23766700	2.94353100	0.28848700
H	10.09377000	3.48596600	0.26075400
H	8.72491000	3.08743600	-0.57751600

Glycine and Proton Sponge

C	1.49353500	0.01302800	0.26143500
C	2.59852600	-0.17260800	-0.64410200
C	1.16502700	1.35722500	0.65908300
C	3.37429600	0.94426100	-1.05405000
C	2.93399800	-1.47442000	-1.10166700
C	0.83001400	-1.16568200	0.74094700
C	1.97146900	2.40819500	0.25275800
N	0.04201300	1.60670800	1.48784200
C	3.08190700	2.20506700	-0.59094100
H	4.20774800	0.77814400	-1.72962200
C	2.23516600	-2.57351900	-0.66151700
H	3.76049800	-1.58323600	-1.79718100
N	-0.22738800	-1.09431500	1.69776400
C	1.18954000	-2.41639700	0.26915800
H	1.71359800	3.41622900	0.56671600
H	0.00801900	2.58074700	1.76870200
H	-0.84642200	1.37325000	1.03148900
H	3.68411700	3.05614200	-0.89411800
H	2.49215400	-3.56791800	-1.01302300
H	-0.19845100	-0.21939000	2.22500700
H	-0.20433500	-1.89307000	2.32465100
H	0.65563000	-3.28955400	0.63483200
O	-2.56364800	-1.30829900	0.35816900
H	-1.69663800	-1.17768800	0.87736500
C	-3.05596400	-0.14360400	-0.03378700
O	-2.53159500	0.94151000	0.19587900
C	-4.37865600	-0.27495000	-0.76075900
H	-4.32269600	-1.14729700	-1.42040100
H	-5.11814700	-0.52077200	0.02246900

N	-4.67239100	0.93148000	-1.52006600
H	-5.66689200	1.03322900	-1.68882600
H	-4.33502600	1.74881700	-1.01856700

Dimethyl Phosphate and PFDA

P	6.17760200	-1.06803100	0.05502900
O	5.37763400	-0.77344600	-1.16771200
O	5.31572200	-1.30312500	1.37998200
O	7.05154800	-2.40656200	-0.01326800
O	7.19579700	0.07343200	0.47086900
H	6.86701100	0.97278800	0.15741500
C	4.19651700	-2.21295100	1.35770900
H	3.67643500	-2.07354800	2.30237000
H	4.55510500	-3.24068200	1.27450500
H	3.53022600	-1.97590100	0.52709400
C	7.89742700	-2.64917300	-1.15393100
H	8.29652800	-3.65305600	-1.02678800
H	8.71558200	-1.92591600	-1.17411500
H	7.31682200	-2.58811200	-2.07615200
N	5.77094500	2.10129400	-0.58192700
H	5.97231500	3.03886500	-0.90882200
C	4.54999300	2.06099600	0.24984200
H	4.68908900	1.32708500	1.04529500
H	4.41853600	3.03129400	0.73060300
C	3.30756000	1.67894000	-0.56490300
H	3.50167700	0.72716900	-1.06719800
H	3.08747100	2.43426200	-1.32174000
C	2.08422000	1.51212300	0.30784100
F	1.74691800	2.69684400	0.91010300
F	2.32836900	0.61335100	1.30906400
H	5.66185600	1.49283500	-1.39133600
C	0.83900100	1.00743700	-0.48174800
C	-0.52130600	1.06227200	0.29327000
F	1.07844200	-0.27387700	-0.85837100
F	0.70959500	1.76095200	-1.60179900
F	-0.33289500	0.64735600	1.56251400
F	-0.95040200	2.34373400	0.31250100
C	-1.64111700	0.16918800	-0.34950400
C	-3.08689200	0.54479800	0.13301700
C	-4.14149800	-0.58533300	-0.13824000
C	-5.62269700	-0.07882600	-0.08101300
C	-6.68500100	-1.21754600	0.05966400
F	-1.59749500	0.30086400	-1.69186000
F	-1.39838800	-1.11986200	-0.02742400

F	-3.47656900	1.66518300	-0.51078300
F	-3.06124600	0.78754100	1.45997300
F	-3.97830900	-1.55596100	0.78577600
F	-3.92483000	-1.11012100	-1.36312800
F	-5.77519300	0.75141300	0.97371700
F	-5.89551900	0.59732900	-1.21414500
F	-6.63264700	-1.77353500	1.27137900
F	-6.48584000	-2.16317100	-0.86509000
F	-7.90200600	-0.69355300	-0.11621200

Dimethyl Phosphate and Proton Sponge

P	-1.95543400	-0.13989500	-0.18451700
O	-1.84641000	-0.12287600	1.29772700
O	-1.07506600	-1.23117800	-0.93298700
O	-3.42641000	-0.44163900	-0.73660600
O	-1.58225100	1.22173900	-0.93011600
H	-0.78298200	1.65832000	-0.58431300
C	-0.95259900	-2.56115800	-0.38312800
H	-0.17673900	-3.04935100	-0.96631900
H	-1.90008300	-3.09392100	-0.48834000
H	-0.65509100	-2.50762300	0.66444400
C	-4.55509900	0.26215600	-0.18340400
H	-5.44067400	-0.20223800	-0.61183600
H	-4.51545000	1.31589800	-0.46781900
H	-4.56641600	0.16561600	0.90367100
C	2.45064000	-2.01978500	0.12795600
C	1.87941900	-1.04226500	0.93377200
C	1.64396400	0.27033600	0.38763000
C	2.07978900	0.52656500	-0.95889700
C	2.64335100	-0.51081400	-1.73914700
C	2.81755100	-1.76089300	-1.19901700
H	2.61619800	-3.00633000	0.54869000
C	1.03397400	1.34992100	1.11385800
C	1.96277900	1.83163800	-1.50513700
H	2.94248400	-0.29522800	-2.75757300
H	3.25498400	-2.55622800	-1.79213600
C	1.43588800	2.85720700	-0.76502200
C	0.95072900	2.60841800	0.53560100
H	2.30457800	1.99951200	-2.51944200
H	1.35597400	3.85429500	-1.18209700
H	0.49010700	3.41215900	1.10054200
N	0.50428600	1.13884200	2.40893400
H	0.19893100	2.01445100	2.81665000
H	-0.30908300	0.51911500	2.35021700

N	1.52488300	-1.37335700	2.23274900
H	2.01168200	-2.16813500	2.61778000
H	1.39769600	-0.59659800	2.86933100

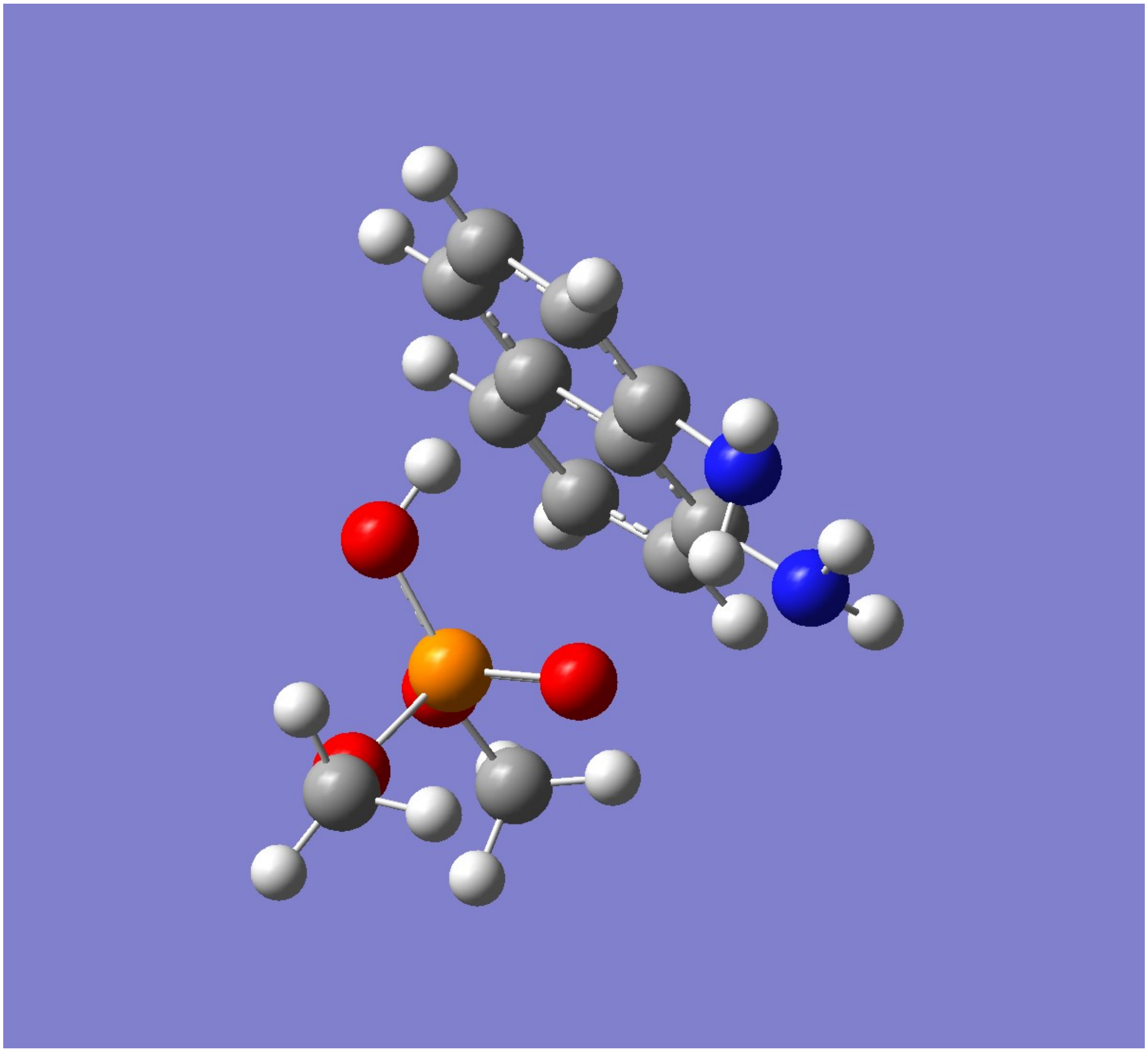

Figure S1. Lowest energy simulated structure of electrostatically bound complex 1,8 bis(dimethylamino)naphthalene and dimethyl phosphate.

Figure S2.

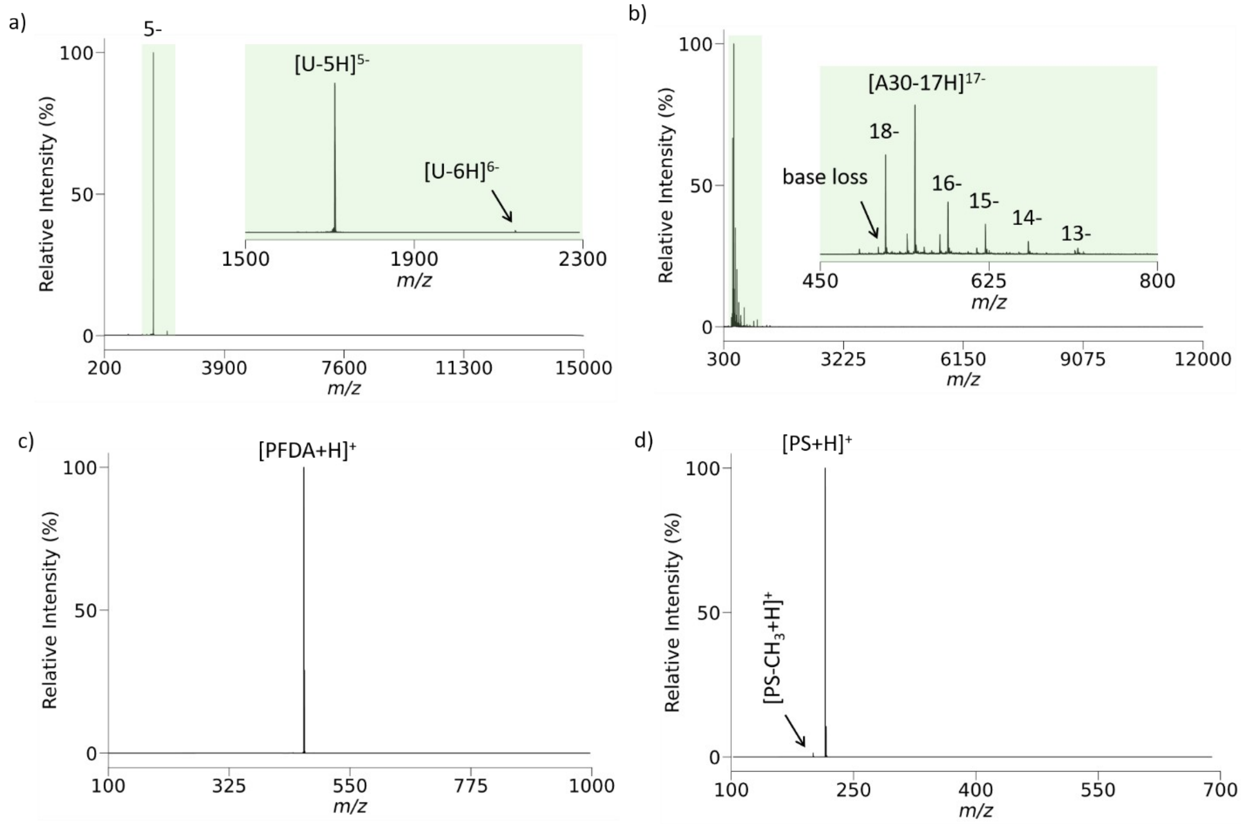

Figure S2. Reactant ion isolation spectra prior to the ion/ion reaction for the data of **Figure 2**. a) The ubiquitin 5- charge state. b) Charge states (13-18)- of A30. c) protonated PFDA. d) protonated proton sponge.

Figure S3.

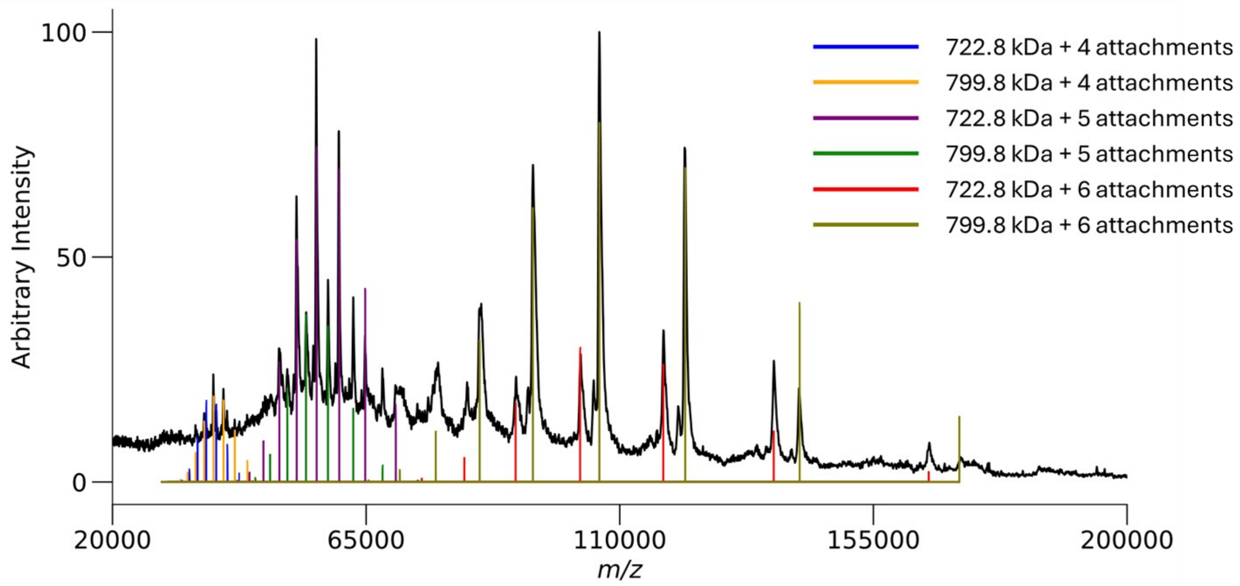

Figure S3. Post ion/ion reaction spectrum following the reactions of the precursor anionic charge states of *E. coli* ribosome 30S particles with the 8+ charge state of bovine ubiquitin.

Attachments	Total Mass (kDa)	Corrected Mass (kDa)
6	824.4	773.0
6	851.2	799.8
5	815.5	772.7
5	842.4	799.6
4	806.5	772.2
4	833.6	799.3

Example calculation: $824.4 - (8.564 \times 6) = 773.01$

Table S1. Each range of charge states resulting from the same number of attachments of the 7+ charge state of ubiquitin to anions of ribosome 30S was subjected to zero-charge deconvolution with the resulting mass corrected for the mass of the respective number of ubiquitin attachments.

Figure S4.

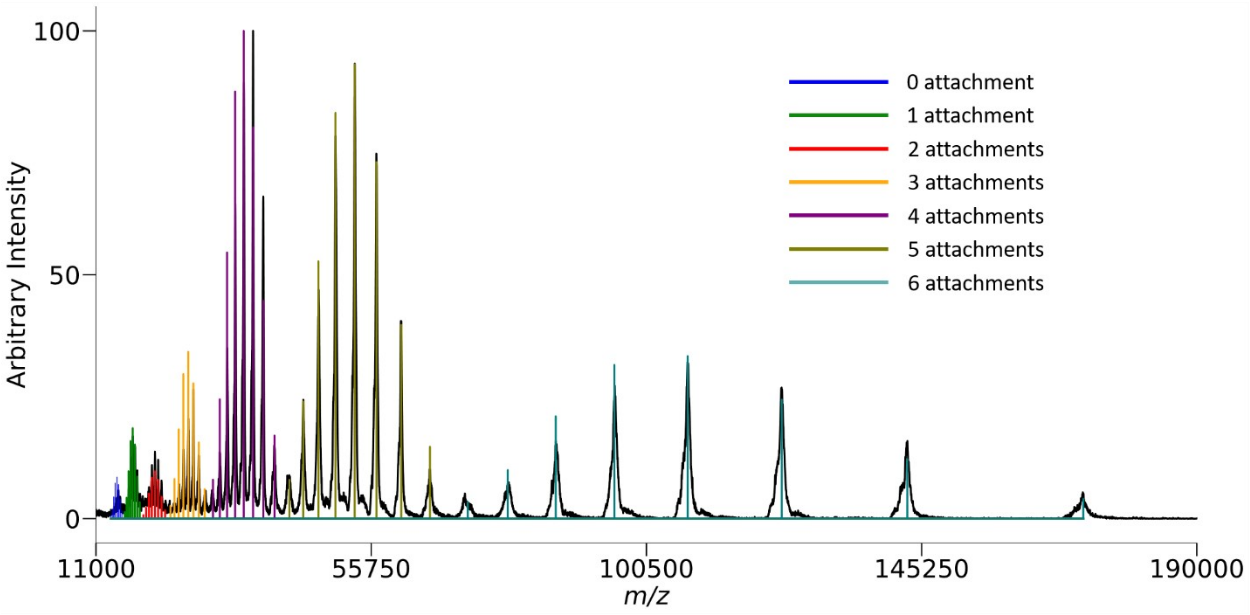

Figure S4. Post ion/ion reaction spectrum following the reactions of the precursor anionic charge states of *E. coli* GroEL with the 7+ charge state of bovine ubiquitin.

Attachments	Total Mass (kDa)	Corrected Mass (kDa)
6	857.8	806.4
5	849.3	806.5
4	840.5	806.3
3	831.9	806.2

Example calculation: $857.8 - (8.564 \times 6) = 806.4$

Table S2. Each range of charge states resulting from the same number of attachments of the 8+ charge state of ubiquitin to GroEL anions was subjected to zero-charge deconvolution with the resulting mass corrected for the mass of the respective number of ubiquitin attachments.

UniDec Parameters	Analytes					
	β-galactosidase		*E. coli* 30S Ribosome		*E. coli* GroEL	
	Pre-IIRXN	Post-IIRXN	Pre-IIRXN	Post-IIRXN	Pre-IIRXN	Post-IIRXN
m/z range	2500-20000	2000-250000	10000-22000	20000-220000	10000-18000	50000-450000
smoothing	0	35	30	30	5	20
Subtract Curved	0	0	0	95	0	0
Acceleration voltage	15	15	0	0	0	0
Binning	5.0 (linear *m/z*)	12 (linear *m/z*)	10 (linear *m/z*)	10 (linear *m/z*)	6 (linear *m/z*)	5 (linear *m/z*)
Charge range	1-60	1-100	1-100	1-50	1-100	1-20
Mass range	5-1000 kDa	5-1000 kDa	500-1000 kDa	500-1000 kDa	300-1000 kDa	5-1000 kDa
Sample Mass	1	1	100	100	1	1
Peak FWHM	6	7	20	400	12	4
Peak Shape	Gaussian	Gaussian	Gaussian	Gaussian	Gaussian	Gaussian
Beta	7	50	20	50	5	5
Charge smooth	1	1	1	1	1	1
Point Smooth	1	1	1	1	1	1
Mass Smooth	0	0	1	1	0	0
# iterations	100	100	1000	1000	100	100
Transformation	Smart	Smart	Smart	Smart	Smart	Smart
Polarity	Negative Mode	Negative Mode	Negative Mode	Negative Mode	Negative Mode	Negative Mode
Peak Detection	500	500	500	500	500	1500
Peak Detect Threshold	0.9	0.1	0.1	0.1	0.05	0.025

Table S3. Parameters used for UniDec zero-charge deconvolution of each analyte pre- and post-ion/ion reaction (UniDec Version 8.0.3 was used for all analytes).
